# Simultaneous HPLC determination of soluble signaling molecules and metabolic status in neuroblastoma cell cultures

**DOI:** 10.14814/phy2.70419

**Published:** 2025-06-19

**Authors:** Natalie Chaves Ferreira, Chiara Gatnau‐Civardi, Miquel Riera‐Codina

**Affiliations:** ^1^ Department of Cellular Biology, Physiology and Immunology, Faculty of Biology University of Barcelona Barcelona 08028 Spain

**Keywords:** cell metabolism, HPLC, inositol phosphates, nucleotides, signaling molecules

## Abstract

Several laboratories have explored the capacity of the anion exchange chromatography method in evaluating distinct forms of inositol phosphates in a single analytical process. We describe a straightforward HPLC method to analyze simultaneously inositol phosphates and nucleotides present in a neuronal cells culture. The method was applied to neuroblastoma cells grown in standard media. The culture has an optimal metabolic state between 45% and 95% of confluence, but there was a rapid metabolic deterioration when the culture density exceeded 100%, which is important to consider when cell stimulation studies are carried out in culture. This method also allows the quantification since 0.5 to 50 nanomoles of nucleotides present in a single confluent culture from a T‐75 flask containing 8 million cells. In addition, cyclic adenosine monophosphate (cAMP) and adenosine monophosphate (AMP) were eluted without overlapping. Therefore, the method has proven to have sufficient sensitivity to determine quantitative changes in nucleotides and inositol phosphates in a sample with low cell density. Moreover, the simultaneous determination of signaling and metabolic molecules allows obtaining a rapid and suitable control of the metabolic status in studies on cell stimulation that should be applicable to other types of cultured cells.

## Graphical abstract

A straightforward HPLC method to analyze simultaneously the major cell inositol phosphates and nucleotides present in the same neuronal cell culture in a single analytical process.

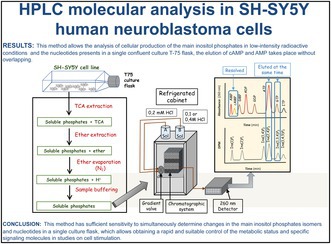

## 1 INTRODUCTION

Soluble forms of inositol phosphate are present at low concentrations in many cells. The most studied form is inositol 1,4,5‐trisphosphate (Ins(1,4,5)*P*_3_) which is present at a very low concentration, but in stimulated cells this inositol form increases quickly and triggers specific processes of signal transduction mediated by Ca^2+^. Since Berridge and Irvine (Berrindge & Irvine, 1984) demonstrated that Ca^2+^ released from the endoplasmic reticulum in smooth muscle cells is mediated by inositol triphosphate (Ins*P*_3_), many studies have investigated this signaling pathway in different cells, such as pancreatoma cells (Horstman et al., 1988), Jurkat cells stimulated by T cell receptor (Guse, Roth, & Emmrich, 1993), neurons and glial cells (Sharp et al., 1999), adrenal chromaffin cells (Poulsen et al., 1995), myeloid cell line HL‐60 (van Delden et al., 1993), rat liver (Rossier et al., 1991), among others.

Most signaling studies use specific Elisa kits to evaluate Ins*P*_3_ level or its effect by measuring intracellular Ca^2+^ level (van Acker et al., 2000; Wilcox et al., 1996). Commercial Elisa tests that measure Ins*P*_3_ have high sensitivity and require a low sample volume, but they only determine one type of inositol phosphate and are not strictly specific, so the signal may be increased if different isomers of Ins*P*_3_ are present. Therefore, it is not useful for signaling pathways in which inositol tetrakisphosphate (Ins*P*_4_) or isomers of Ins*P*_3_ other than Ins(1,4,5)*P*_3_ are involved (Yang et al., 1999).

Cytoplasmic soluble forms can be phosphorylated by specific inositol kinases and hydrolyzed by phosphatases (Ho et al., 2002; Irvine, 2005; Irvine & Schell, 2001; Letcher et al., 2008; Loss et al., 2013; Michell, 2008) giving rise to different inositol forms. These forms are of interest as possible signaling molecules, but the previously referred methods cannot evaluate them simultaneously if they are not complemented with chromatographic determinations (Horstman et al., 1988); many of them were reviewed recently (Maffucci & Falasca, 2020).

Some studies have explored the role of inositol phosphates different from Ins*P*_3_. The production of different Ins*P*_4_ isomers or inositol pentakisphosphate (Ins*P*_5_) has been related to insulin action (Renström et al., 2002), hemopoietic cell differentiation (Mountford et al., 1994), neuronal differentiation (Loss et al., 2013), lipopolysaccharide stimulation in macrophage cells (Casals et al., 2002) among others. Increases in some inositol phosphates could inhibit different cellular processes such as the phosphoinositol 3‐kinase (PI3K) signaling pathway (Eckmann et al., 1997), neutrophil chemotaxis (Jia et al., 2007), chloride flux in epithelial cells (Xie et al., 1996) or in general alter energy metabolism (Chatree et al., 2020). Furthermore, it has been known for a long time that inositol 1,3,4,5,6‐pentakisphosphate (Ins(1,3,4,5,6)*P*_5_) isomer interacts with avian hemoglobins (Bartlett, 1980; Isaacks & Harkness, 1980; Riera et al., 1991) and more recently, this inositol has been described as having a role in mammalian signaling pathways, for example, in promoting apoptosis in cancer cells through the PI3K/protein kinase B (AKT) pathway (Piccolo et al., 2004). Some reviews about the role of highly phosphorylated inositols indicate that these inositol isomers have been poorly explored by chromatography in animal cells and tissues (Burton et al., 2009; Chatree et al., 2020; Ho et al., 2002; Letcher et al., 2008; Maffucci & Falasca, 2020; Matejuk & Shamsuddin, 2010; Michell, 2008), possibly due to a lack of available direct and accurate analytical methods.

In addition to the determination of inositol isomers, the measurement of the nucleotide levels allows monitoring the status of cell cultures through the identification of their metabolic status (adenylate energy charge) and some indication about the degree of apoptosis with adenosine triphosphate (ATP)/guanosine triphosphate (GTP) ratio (Bereiter‐Hahn et al., 1998; Bradbury et al., 2000; Prete et al., 2008). It is of great importance for studies of cell signaling to follow the metabolic status of the culture after activation with an agonist because the density and conditions of the culture could induce metabolic changes in the cells (Bereiter‐Hahn et al., 1998).

Previously, we found interesting similitudes in inositol phosphates of bird and crocodilian erythrocytes of interest in comparative studies (Villar et al., 2003). However, this chromatographic method does not resolve cAMP from AMP and requires a large amount of sample to determine accurately the minor soluble inositol phosphates (King et al., 2010).

Here, we propose a method that is appropriate for cell cultures, which allows us to evaluate simultaneously several signaling molecules and the cellular metabolic status. We used the SH‐SY5Y neuroblastoma cell line because they grow in standard culture media (Dulbecco's, etc.) like many other cells, they can be easily differentiated and are of great interest for broad neurophysiological studies. (Kovalevich et al., 2021). This method is straightforward and enables an appropriate resolution to elute cAMP separately from metabolic nucleotides. It is also efficient enough for the determination of low levels of inositol phosphates by incubation with myoinositol labeled with tritium (^3^H‐myo‐inositol) at low intensity.

## 2 MATERIALS AND METHODS

The main equipment and reagents used in the method are shown in Table 1.

**TABLE 1:** List of equipments, materials, and reagents used in this method.

Equipament/reagent/material	Stock initial concentration	Catalog number	Vendor, country
Pharmacia LKB 2150 HPLC Pump	–	–	Amersham plc, Sweden
Pharmacia PSV‐50 solenoid valve and	–	–	Amersham plc, Sweden
Pharmacia LKB GradiFrac fraction collector	–	–	Amersham plc, Sweden
Injection valve: 7125 LC sample injector	–	–	Rheodyne, USA
RESOURCE Q anion exchange chromatography column	–	–	Amersham plc, Sweden
Milli‐Q purification system	–	–	Merck‐Millipore, France
UV–Vis SPD‐10A Shimadzu detector	–	–	Shimadzu, Japan
Tri‐Carb 2100TR Liquid Scintillation Counter	–	–	Packard, USA
PC‐Logger 3100	–	–	Intab, Sweden
Grams/AI. Versión 9.1	–	–	Thermo Fisher Scientific Inc., USA
GraphPad Prism 10	–	–	GraphPad‐Dotmatics, USA
^3^H‐myo‐inositol	1 μCi/mL	NET114A001MC	PerkinElmer, Spain
ATP	1 mM	A7699‐1 g	Sigma‐Aldrich Quimica, USA
ADP	1 mM	01897‐250 mg	Sigma‐Aldrich Quimica, USA
AMP	1 mM	01930‐5 g	Sigma‐Aldrich Quimica, USA
Antibiotic Antimycotic Solution	100x	A5955‐100 mL	Sigma‐Aldrich Quimica, USA
cAMP	1 mM	D0260‐5 mg	Sigma‐Aldrich Quimica, USA
Diethyl ether	99.9%	309,966‐1 L	Sigma‐Aldrich Quimica, USA
DMEM High Glucose	1×	L0104‐500 mL	Biowest, France
DPBS w/Ca^2+^/Mg^2+^	1×	L0625‐500 mL	Biowest, France
DPBS w/o Ca^2+^/Mg^2+^	1×	L0615‐500 mL	Biowest, France
FBS	‐	F7524‐50 mL	Sigma‐Aldrich Quimica, USA
GTP	1 mM	G8877‐25 mg	Sigma‐Aldrich Quimica, USA
GDP	1 mM	G7127‐25 mg	Sigma‐Aldrich Quimica, USA
GMP	1 mM	G8377‐500 mg	Sigma‐Aldrich Quimica, USA
Hydrochloric acid	37%	320,331‐500 mL	Sigma‐Aldrich Quimica, USA
IMP	1 mM	57,510‐5 g	Sigma‐Aldrich Quimica, USA
Millex‐AP filter	–	SLAP02550‐25 mm	Merck‐Millipore, Germany
Trichloroacetic acid	20%	252373.1611‐1 L	Panreac AppliChem, Spain
Trypsin–EDTA solution	0.25%	T4049‐100 mL	Sigma‐Aldrich Quimica, USA
T‐25 flask	–	169,900	ThermoFisher Scientific, USA
T‐75 flask	–	156,800	ThermoFisher Scientific, USA
Ultracel‐30 regenerated cellulose membrane	–	UFC803096‐96 u	Merck‐Millipore, Germany

### 2.1 Cell culture

For the analytical procedures, the SH‐SY5Y cell line was used, a neuroblastoma originally isolated from a biopsy of a metastatic bone tumor taken from a 4‐year‐old female (Kovalevich et al., 2021). Specifically, this epithelial cell line was purchased from Eucellbank (catalog n°0057‐HNCL), a cell bank of the University of Barcelona. SH‐SY5Y cells express the erythropoietin receptor and are widely used for in vitro experiments to study the signaling triggered by erythropoietin (EPO) in the nervous system. Neuroblastoma cells were cultured in T‐25 and T‐75 flasks with DMEM medium (Dulbecco's Modified Eagle's Medium) supplemented with 10% fetal bovine serum (FBS) and 1% antibiotic antimycotic solution at a temperature of 37°C, with 5% CO_2_ and 21% O_2_. Subcultures were performed when 95% confluence was reached, using 0.25% Trypsin/EDTA and phosphate‐buffered saline (PBS) without Ca^2+^/Mg^2+^, while for normal washing every 2 days, PBS with Ca^2+^/Mg^2+^ was used.

To evaluate inositol phosphates, cells were incubated with ^3^H‐myo‐inositol at a concentration of 4 μCi/mL of culture in T‐75 flasks. The best condition to obtain a suitable incorporation and metabolization of the labeled inositol was to incubate cells during 24–36 h from an initial confluence of 50%–60% (from 4.2 × 10^6^ cells to 5 × 10^6^ cells per T‐75 flask).

The SH‐SY5Y cell line was cultured in T‐25 flasks when only nucleotide determination was needed. For the complete analysis of nucleotides and inositol phosphates, T‐75 flasks were used The number of cells when the culture just reached confluence was approximately 3 million for a T‐25 flask and 9 million for a T‐75 flask.

### 2.2 Trichloroacetic acid extraction

A confluent cell culture performed in T‐75 flasks was washed gently three times with 80 mL of PBS with Ca^2+^/Mg^2+^ to maintain cells adhered to the bottom of the flask. After washing, medium was aspirated and 3 mL of distilled water and 6 mL of 10% trichloroacetic acid (TCA) were added and the flasks were placed on ice in an orbital agitator for 45 min to lyse cells and to recover cell phosphates. The content of each flask was aspirated with a syringe and directly filtered through a Millex‐AP filter into 50 mL pyrex tubes. This method allows for the rapid elimination, in one step, of membrane residues and non‐soluble cell compounds. It was tested that no inositol phosphates or nucleotides were retained in the filter. As nucleotides triphosphate hydrolyze quickly at low pH, the TCA was promptly removed from the aqueous solution by four successive extractions with diethyl ether (TCA extract volume/ether volume ratio of 1:3), shaking each tube in a vortex and removing the upper solution, containing ether and TCA, in each extraction. By this process, the amount of TCA in the aqueous solution is reduced by 25 times; more extractions do not reduce the remaining TCA. Ether was evaporated by bubbling with nitrogen using an 8‐port glass gas distributor. Finally, the solution was ultra‐filtered with 30.000 Da filter and diluted with 10 mM phosphate buffer, pH 7.5 at a ratio of 1:1 to neutralize the remaining TCA. It is important to maintain the ratio of phosphate buffer/extract because the amount of phosphate buffer used subsequently influences the nucleotides elution time (see Results). In this way, the final pH of the solution was 6–7 and samples could be maintained at −80°C for a month without appreciable hydrolysis of ATP. It is important to extract quickly the TCA from the samples. Trials made with ATP resulted in hydrolysis of 20%–30% when it was maintained for a month at −20°C in 10% TCA solution.

If only nucleotides are being assessed, confluent T‐25 cultures can be used with the following modifications: T‐25 flasks are washed with 20 mL of PBS with Ca^2+^/Mg^2+^; after complete PBS aspiration, 1 mL of distilled water and 2 mL of 10% TCA are added, and they are filtered through a Millex‐AP filter to a 30 mL Pyrex tubes.

### 2.3 Chromatographic conditions

In the method, the eluents used were 0.2 mM hydrochloric acid (HCl) (A solution) and 0.1 M HCl (B solution). The elution was carried out at a 1 mL min^−1^ flow rate, increasing the concentration of mobile phase B as shown in Table 2. Higher flow rates tested (data not shown) reduced the detection signal of the molecules and made the elution peaks broader. Chromatographies were developed in a column temperature lower than room temperature (16°C–18°C). Several gradients and concentrations of B eluent were tested in order to reach a better nucleotide separation and a higher signal‐to‐noise ratio (more sensitivity). These conditions allow an optimal separation of AMP and cAMP but eluted only inositol phosphates, inositol disphosphate (Ins*P*_2_) and Ins*P*_3_. The important thing is to maintain a low gradient slope to elute nucleotides and poorly phosphorylated inositols and increase the gradient slope for highly phosphorylated inositols. To elute inositol phosphates with higher phosphorylation, it is required to apply a B solution with a higher HCl concentration (0.4 M).

**TABLE 2:** Description of the gradient profile used for any B solution.

Time	B solution
(min)	(%)
0	0
4	2
7	5
9	8
12	9
13,5	11
19	17
31	28
48	65
50	100

*Note*: Chromatographies were usually performed at a flow rate of 1 mL min^−1^.

After elution, the nucleotides were measured by their absorbance at 260 nm. Soluble inositol phosphates were collected separately in 1 min fractions, and the tritium radioactivity was measured by mixing 0.5 mL of each fraction with 3.5 mL of scintillation cocktail and counted to obtain DPMs. If the inositol amount in the sample is very low, the entire volume of each fraction of 1 mL is mixed with 8 mL of scintillation cocktail. This almost duplicates the DPMs signal.

### 2.4 Grams software application to evaluate and optimize peak areas

The elution shape is characteristic of each nucleotide. Thus, nucleotide patterns registered were analyzed using a Grams software (Grams/AI. Versión 9.1). To adjust the parameters that fit better with any nucleotide shape, we found the following Gaussian values for Full Width at Half Height (FWHH): 0.28 for AMP, cAMP, and ADP; 0.55 for ATP; 0.45 for GDP; and 0.60 for GTP. These values allow obtaining the best integration area for each nucleotide present in the samples. This characterization is also useful if, in the samples, any nucleotide appears to overlap with any other molecule. In this case, the Peak Fitting function is used, which enables the overlapped plot to fit into two peaks. The above FWHH parameters defining each involved nucleotide allow a better estimation of the actual area.

### 2.5 Statistical analysis

The statistical analysis for the confluence study was done using a GraphPad Prism 10. Data were expressed as the mean ± standard deviation, and statistical significance was determined by ANOVA one‐way test. Differences were considered statistically significant at *p* < 0.05.

## 3 RESULTS

### 3.1 Standard calibration curves and molar extinction of the main nucleotides eluted

The nucleotide molar extinction coefficient (*ε*) varies for each nucleotide and for the pH (Cohn & Hughes Jr, 1960), being slightly lower in acid medium. Trials done in our laboratory shown in Table 3 gave a ratio *ε*_H2O_/*ε*_HCl_ at 0.05 M of 1.03, 1.05, 1.07, 1.01 and 1.08 for ATP, ADP, AMP, GDP, and GMP, respectively. This pH effect is less evident when the nucleotide is more phosphorylated because the accumulation of phosphates produces a buffering effect on the nucleobase. Then, we calculated the specific molar extinction of each nucleotide just at the pH that is eluted by the method.

**TABLE 3:** Molar extinction coefficient (*ε*) for different nucleotides measured in water solution and 0.05 M HCl.

Nucleotide	*ε* value in different solutions	
	H_2_O	HCl
ATP	13.0	12.6
ADP	13.2	12.6
AMP	13.6	12.7
GDP	8.4	8.3
GMP	9.9	9.2

*Note*: Chromatographies were usually performed at a flow rate of 1 mL min^−1^.

Table 4 shows the accurate values of the linear regression–the intercept was forced to be 0—of nucleotides eluted when a B solution of 0.4 M HCl and 0.1 M HCl are applied. For each nucleotide, 50, 25, 10, 5, 2.5, 1, and 0.5 nmoles were applied to calculate the linear regression. The regression slope of the nucleotides also depends slightly on the HCl concentration of the B solution used, as well as how many phosphate groups are contained in the nucleotides. The slope obtained for each nucleotide is lower when the number of phosphate groups increases, because they are eluted and consequently measured at lower pH. Moreover, the slope variance is dependent on the nucleotide type; for instance, adenine nucleotides have higher slopes than guanine ones. As a result, the analytical method is more sensitive to low‐phosphorylated adenine nucleotides, meaning that the sensitivity is higher for AMP or cAMP compared to ATP or GTP. We presented values for adenine and guanine nucleotides in the conditions proposed in the present study; if other nucleotides are used, it would be necessary to calculate the correspondent slope.

**TABLE 4:** Linear regression of the nucleotides tested by the chromatographic method. This table shows the exact values of the linear regression obtained from the absorbance peak area at 260 nm as a function of the μmoles of nucleotides applied when the chromatography was developed in a gradient of B solution of 0.1 M HCl and 0.4 M HCl.

Nucleotide	Chromatographic eluting solution (HCl)					
	0.1 M			0.4 M		
	Eluting time (min)	Slope	*R* ^2^	Eluting time (min)	Slope	*R* ^2^
AMPc	9.6	11,013	0.999	5.3	8017	0.976
AMP	7.2	13,582	0.999	5.3	9355	0.998
ADP	16.0	11,790	0.999	9.4	9075	0.995
ATP	35.7	10,641	0.996	16.92	8625	0.999
GMP	12.3	10.663	0,999	6.47	9672	0.976
GDP	26.6	9089	0.999	13.13	8355	0.999
GTP	43.9	7522	0.996	20.79	6502	0.996
IMP	12.7	7696	0.999	7.63	6585	0.999

*Note*: Chromatographies were usually performed at a flow rate of 1 mL min^−1^.

### 3.2 Range of sensitivity

**FIGURE 1:**
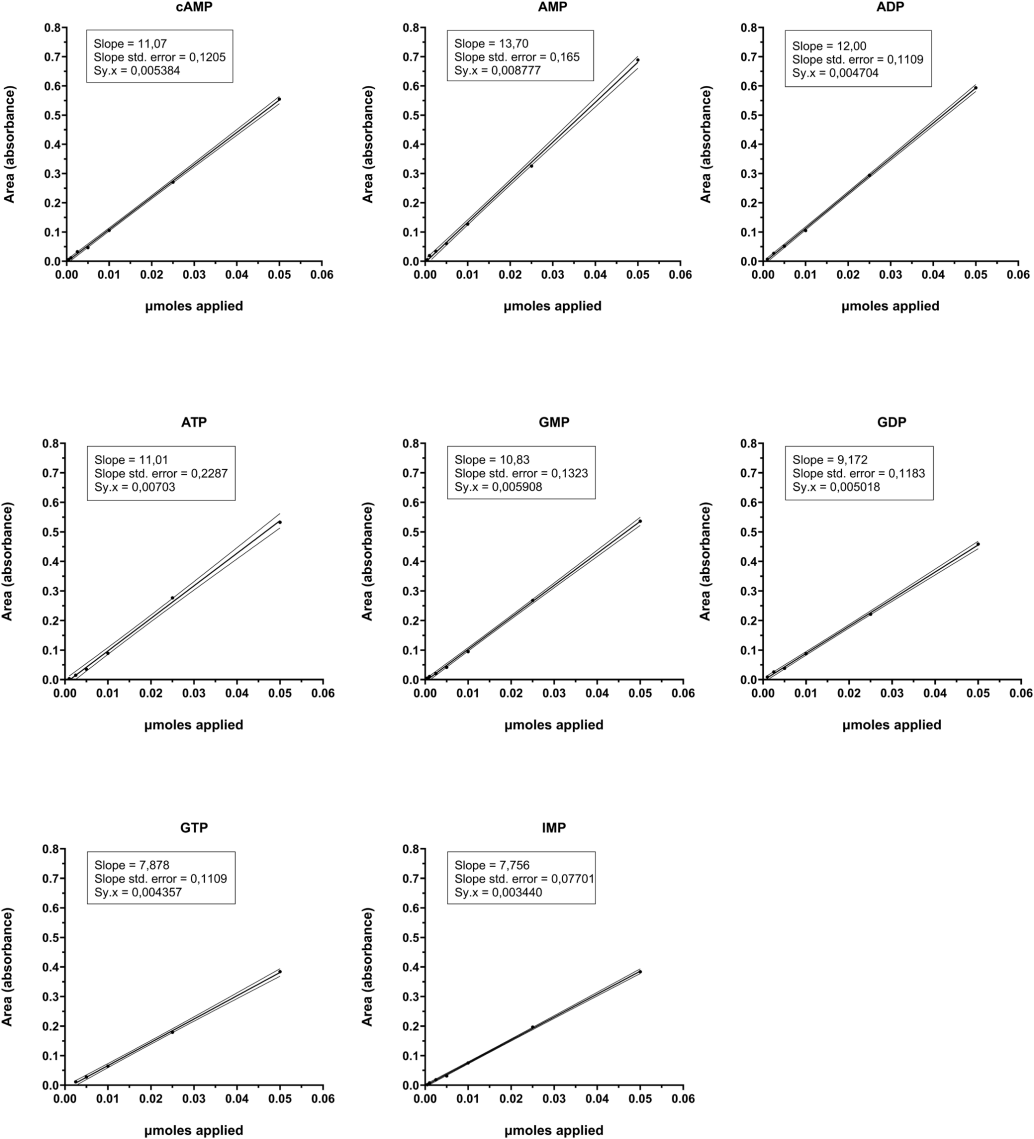
Linearity and confidence intervals (*p* < 0.05) of the different nucleotides eluted at 0.1 M HCl. Data were obtained from 50, 25, 10, 5, 2.5, 1, and 0.5 nmoles applied. The slope, the standard error of the slope, and the standard deviation of the residuals are indicated for each nucleotide in the figure.

**FIGURE 2:**
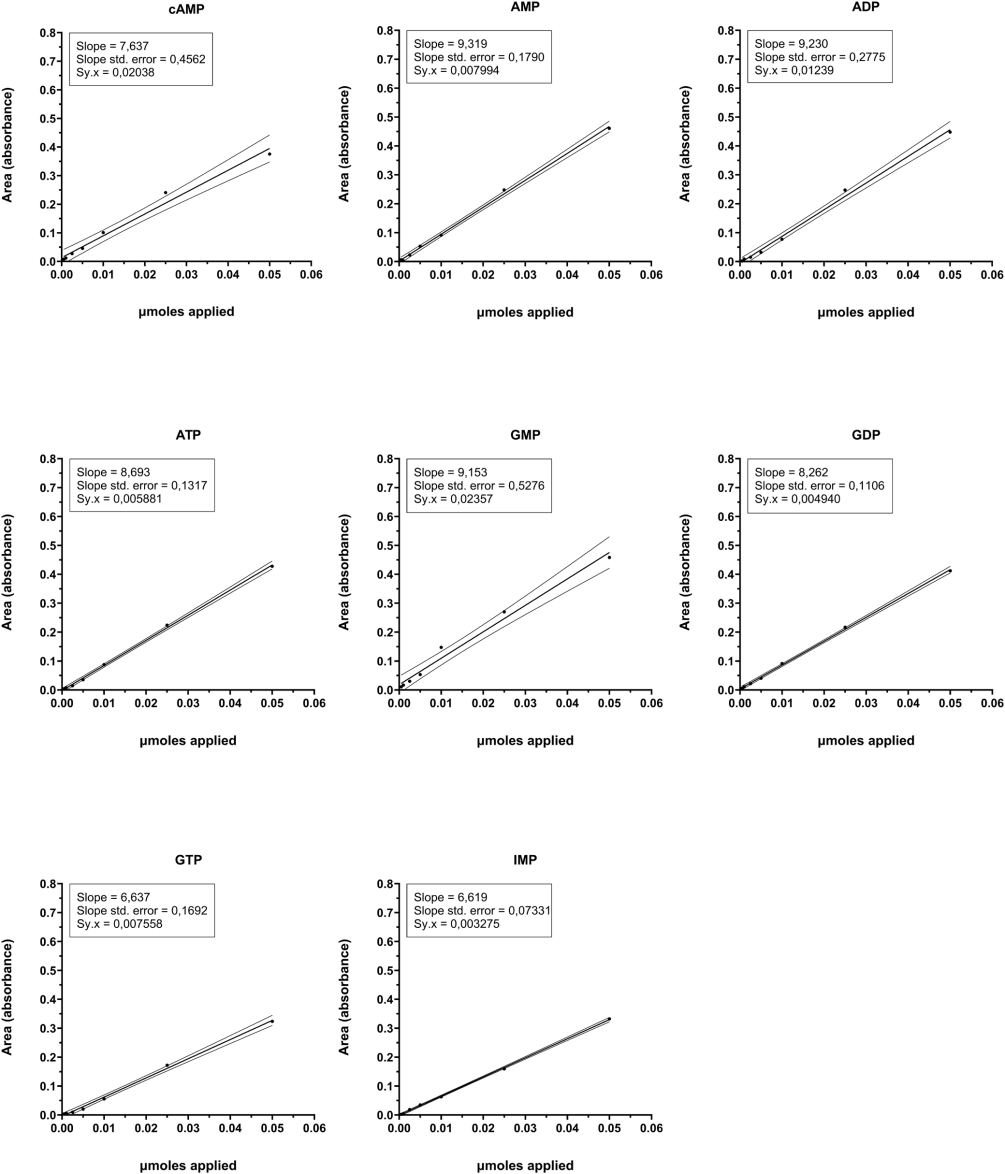
Linearity and confidence intervals (*p* < 0.05) of the different nucleotides eluted at 0.4 M HCl. Data were obtained from 50, 25, 10, 5, 2.5, 1, and 0.5 nmoles applied. The slope, the standard error of the slope, and the standard deviation of the residuals are indicated for each nucleotide in the figure.

The relationship between the amount of nucleotide applied and the pick area obtained was very linear from 0.5 nmoles to 50 nmoles for all nucleotides tested. Figures 1 and 2 show the linearity and confidence intervals (*p* < 0.05) obtained for 50, 25, 10, 5, 2.5, 1, and 0.5 nmoles applied of the different nucleotides eluted at 0.1 or 0.4 M HCl. The slope, the standard error of the slope, and the standard deviation of the residuals were indicated for each nucleotide.

As can be seen in those figures, the confidence intervals are very narrow. This indicates that the analysis is very linear between the interval of 0.5 and 50 nmoles for all nucleotides tested. Then, the limits of detection are at least of 0.5 nmoles to the minimum limit and 50 nmoles to the maximum limit, proving to be a very sensitive method for a wide range of applied nucleotides. For this reason, the method allows evaluating amounts of nucleotides related to a factor of 100. This is very useful for simultaneous characterization of metabolic and signaling status of the cell culture, since the analytical method is suitable for simultaneously analyzing high ATP and low cAMP concentrations in a specific sample.

### 3.3 Effect of phosphate buffer on nucleotide elution time

The concentration of phosphate buffer used for sample neutralization had a slight effect on the nucleotide elution time; this effect was mainly observed on monophosphate nucleotides. The assays performed in our laboratory, shown in Table 5, indicate that the presence of 18 mM phosphate buffer produces a delay of almost 3 min for the monophosphate nucleotides tested, while the delay is reduced to less than 1 min for the di‐and triphosphate nucleotides. In the method, the amount used (5 mM phosphate buffer) has a very short impact on the elution time, but it is important to take this effect into account if more volume of buffer is used in an experiment.

**TABLE 5:** Effect of the concentration of the phosphate buffer used for sample neutralization on the nucleotide elution time obtained by this HPLC method.

Phosphate buffer concentration (mM)	Elution time (min)					
	AMP	GMP	ADP	GDP	ATP	GTP
0	6.87 ± 0.36	11.77 ± 0.55	16.27 ± 0.54	25.68 ± 0.53	35.52 ± 0.50	44.03 ± 0.48
6	8.12 ± 0.24	13.28 ± 0.10	16.67 ± 0.15	26.22 ± 0.15	36.00 ± 0.18	44.37 ± 0.14
12	8.93 ± 0.49	13.93 ± 0.29	16.80 ± 0.26	26.38 ± 0.29	36.13 ± 0.29	44.47 ± 0.23
18	9.65 ± 0.38	14.45 ± 0.10	16.92 ± 0.10	26.52 ± 0.13	36.27 ± 0.16	44.58 ± 0.10

*Note*: The elution flow was adjusted to 1 mL min^−1^ and B solution was 0.1 M HCl. Data are means ± SD. *n* = 3 biological replicates.

### 3.4 Gradient optimization for cAMP and AMP resolution

**FIGURE 3:**
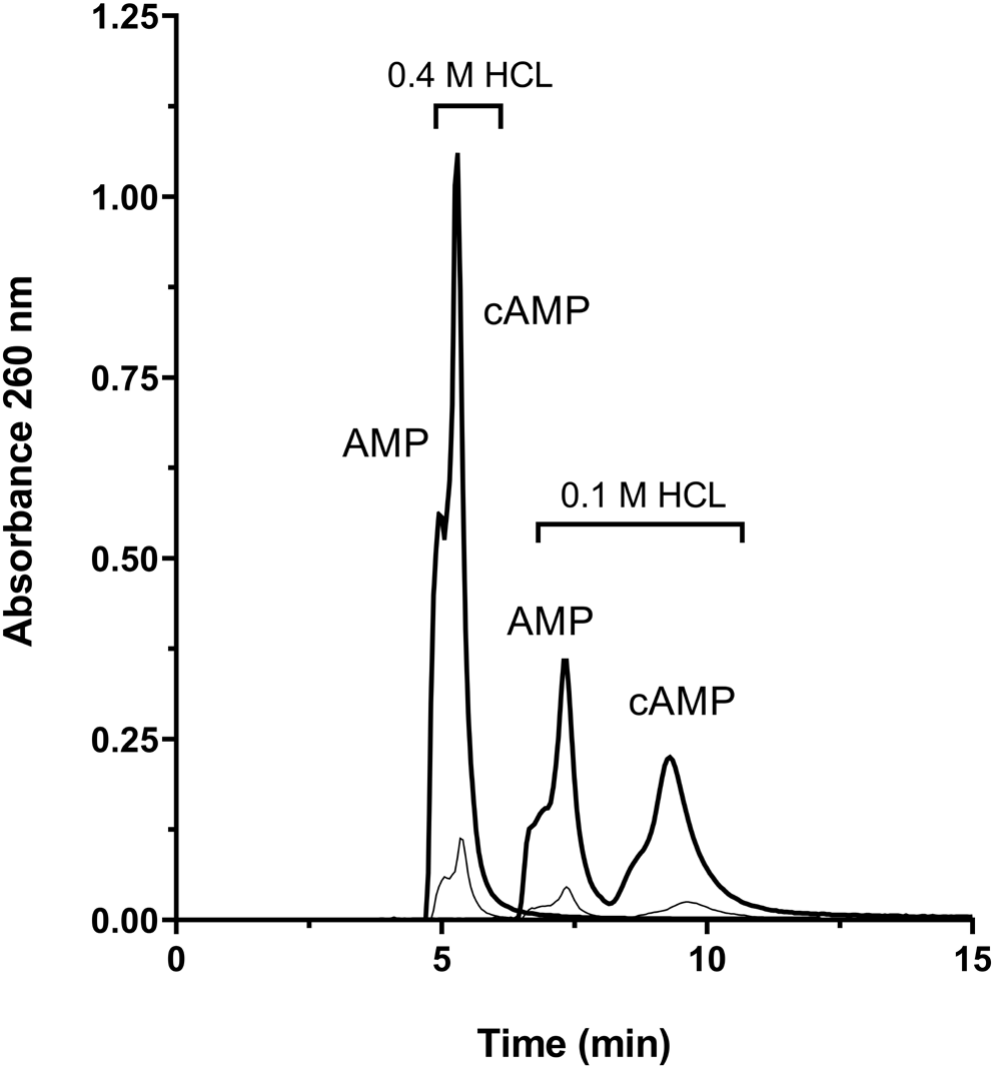
Chromatographic separation of AMP and cAMP at different B solutions. We show here the chromatograms obtained when two extreme levels of B solution (0.4 M HCl and 0.1 M HCl) were used. In each case, two concentrations of nucleotides were applied (2 nmoles, narrow line; and 20 nmoles, wide line). Each chromatography was performed at a flow rate of 1 mL min^−1^.

The concentration of B solution used in previous studies (Casals et al., 2002; Guse, Roth, & Emmrich, 1993) does not give a good resolution for AMP and cAMP. It is important for signaling studies to resolve both nucleotides in the same analytic process. We tested different B buffer concentrations and the best B concentration was 0.1 M HCl, which resolves appropriately both nucleotides and has enough strength to elute GTP and inositol tetrakisphosphates in the same chromatographic process. The chromatograms obtained under the proposed conditions and using either 0.1 M HCl or 0.4 M HCl B solution, in a mixture of AMP and cAMP, are shown in Figure 3. Using a B solution of 0.1 M HCl completely resolved both nucleotides for both low (2 nmol) and high (20 nmol) amounts of standard applied.

### 3.5 Effect of culture confluence on cell metabolism

In order to apply the method to assess the metabolic state of cultured cells and evaluate optimal culture conditions, we analyzed the nucleotide content in SH‐SY5Y cells cultured at increasing levels of culture confluence, from 25% to 150%. Cultures were grown in T‐25 or T‐75 flasks. T‐25 flasks were used for high confluences (95%–150%), while for low confluences, cells were grown in T‐75 flasks. When the confluence of SH‐SY5Y cells reached the desired value, the nucleotides were extracted from the cells and the extracts were analyzed as indicated in the methodology. The different culture confluences were obtained by trypsinizing a 100% confluent culture and seeding a volume of cell suspension appropriate to obtain the desired confluency 2 days later. Since these cells grow at approximately 30% per day under the culture conditions described in the methods, the confluence to seed 2 days before the analysis was calculated as follows:

$$\mathrm{SC}=\mathrm{AC}/{\left(1,3\right)}^{2}$$

SC = seeding confluence 2 days before analysis.

AC = confluence desired for analysis.

**FIGURE 4:**
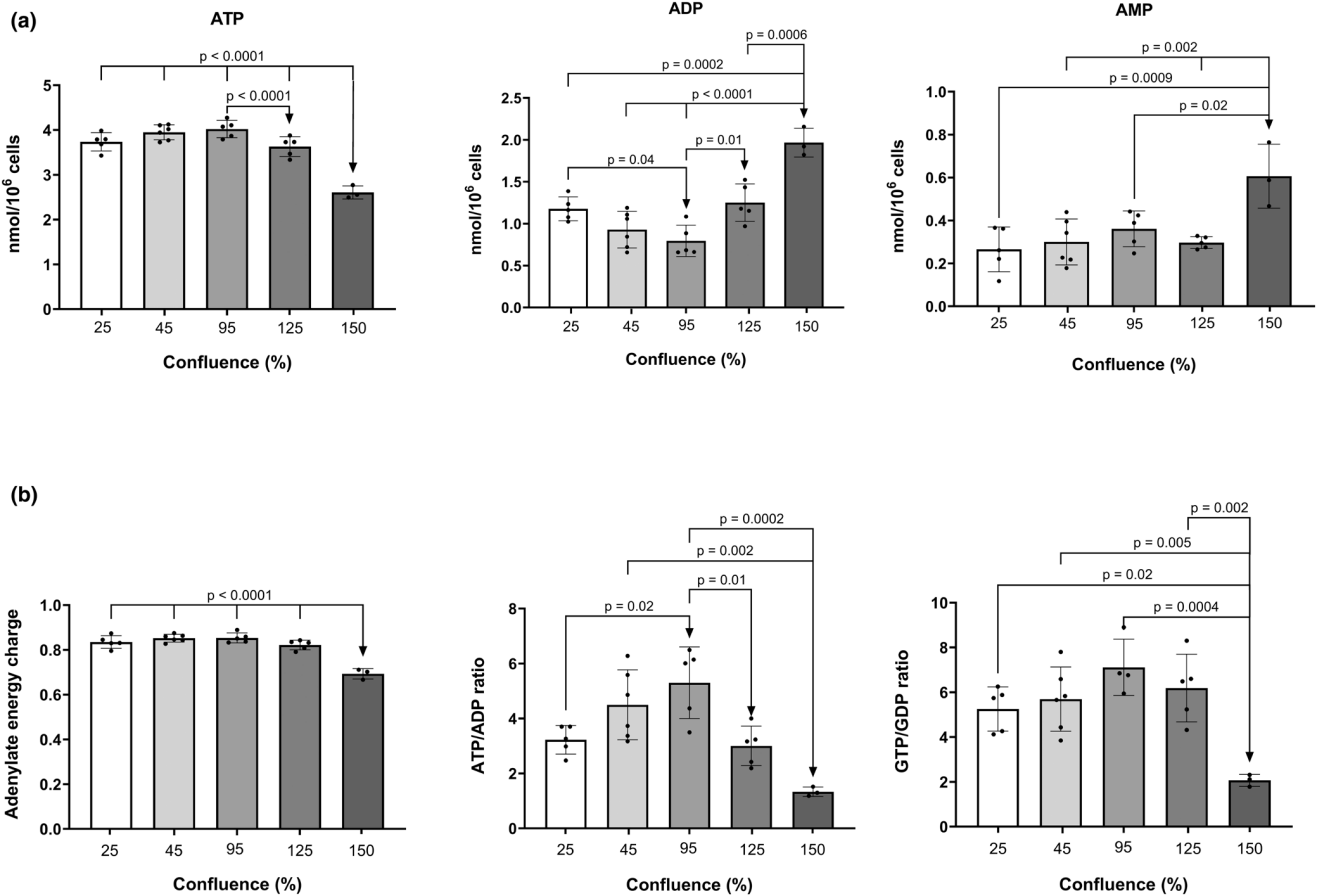
Cellular amount of the main adenosine nucleotides at different culture confluences. The levels of (a) adenine nucloetides and (b) adenylate energy charge and nucleotide ratios were analyzed in SH‐SY5Y cell line. The chromatographic analyses were done in the proposed conditions shown in methods. Statistical analyses were performed by one‐way ANOVA followed by Tukey's post hoc test. A value of *p* > 0.05 was considered as statistically significant. Data was shown means ± SD (*n* = 6 biological replicates).

For example, to achieve 95% confluence, a 100% confluent T‐25 culture is resuspended in 2 mL of medium and then seeded with 1.1 mL (56% confluency) of the suspension into another T‐25 culture. The initial confluence will therefore be approximately 56%, and on the second day of culture, 95%–100%. For approximately 150% confluence, a culture at 89% confluence was incubated for 2 days prior to analysis. The main results are shown in Figure 4. For adenosine nucleotides (Figure 4a), the cellular ATP concentration was maintained at the highest levels (3.7–3.8 nmol/million cells) for confluences between 45% and 95%. Lower confluences tend to slightly decrease cellular ATP content. However, when the confluence of cultures exceeded 100% (from 125% to 150%), a rapid and statistically significant decrease in this phosphate was observed. These trends were opposite to those observed in ADP, which can be more clearly evidenced when it is expressed as the ATP/ADP ratio (Figure 4b).

These results indicate a metabolic hydrolysis of ATP, suggesting a metabolic depletion of the cells at confluences higher than 100%. The main indicators of the metabolic status, adenylate energy charge ([ATP] + ½ [ADP]/[AMP] + [ADP] + [ATP]) and nucleotide ratios, are shown in Figure 4b. No significant differences are found among the confluences from 25% to 125%, but a significant decrease takes place for confluences higher than 125%. These results confirm that when confluence is above 100%, cells suffer a metabolic depletion possibly due to a limitation in oxygen and glucose availability.

### 3.6 Inositol phosphates radiolabeled quantification

**FIGURE 5:**
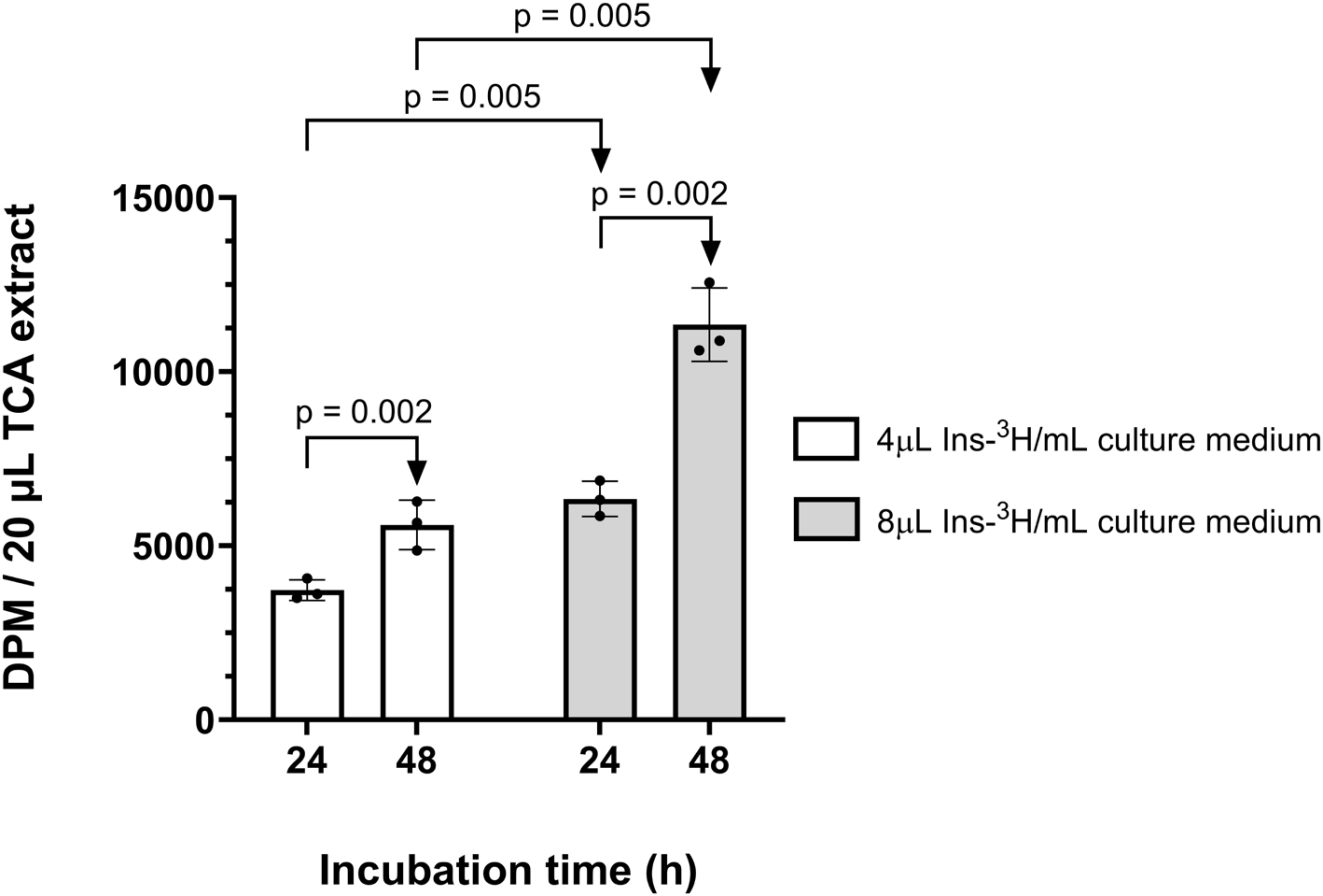
Incorporation rate of Ins‐^3^H in a SH‐SY5Y culture. The figure shows the incorporation rate of Ins‐^3^H in the SH‐SY5Y cell line as a function of the incubation time and radiolabel dose to optimize the maximum signal for the minimum volume of Ins‐^3^H. Cells were cultured in 12 well‐plates for 24 and 48 h at the 4 μL and 8 μL Ins‐^3^H/mL culture medium. The cell culture confluence was 40% at the beginning, and it reached 70% after 48 h. Data was shown as means ± SD (*n* = 3 biological replicates).

In order to minimize radioactive exposure, Ins‐^3^H incorporation tests were performed on SH‐SY5Y cells in culture. In Figure 5 it can be seen that doubling the dose of radioactivity doubles the incorporation rate, but the same effect is achieved if the incubation time with radioactivity is duplicated. In our experience, a dose of 4 μL of ^3^H‐myo‐inositol in culture medium is sufficient to determine changes in cellular levels of inositols.

**FIGURE 6:**
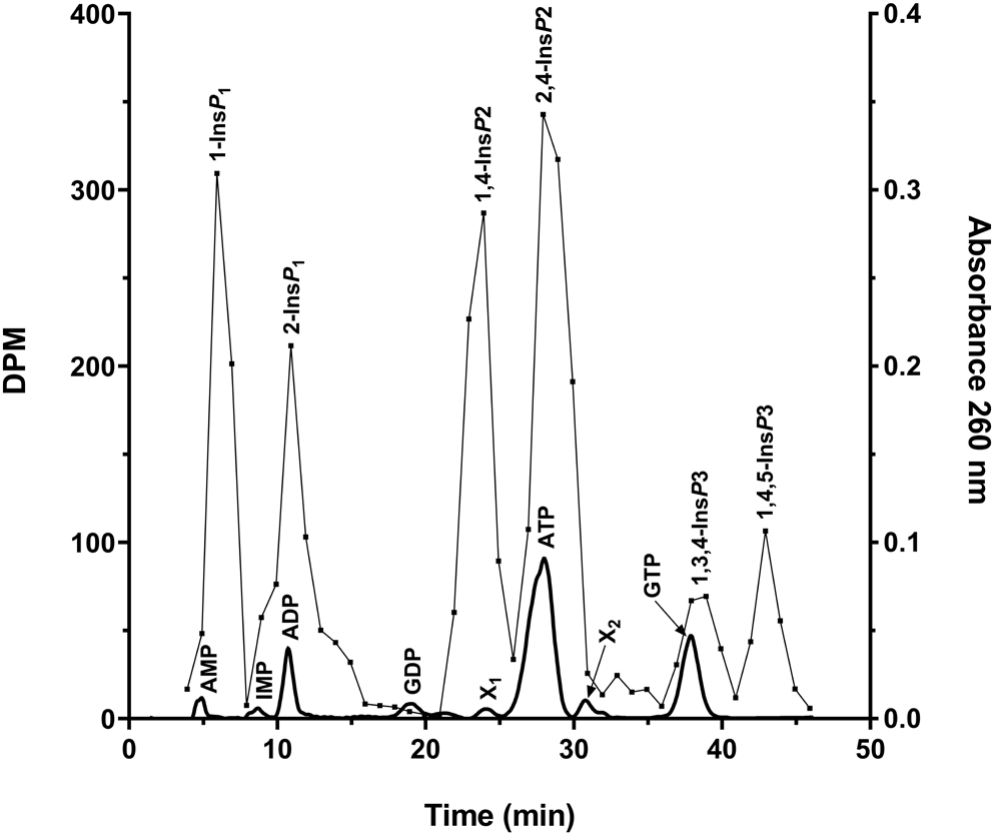
Chromatogram obtained by radioactivity (inositol phosphates) and absorbance at 260 nm (nucleotides) in the same cell extract. The cells were cultured in a T‐75 flask. 24 h before confluent extraction, Ins‐^3^H was added to the culture at a dose of 6 μCi/mL of culture medium. The extraction of organic phosphates was carried out as indicated in the methods at a confluence in the flask of around 90%. The chromatography was performed on a Resource Q column with a gradient of 0.1 M HCl and a flow rate of 2.75 mL min^−1^. Narrow lines indicate disintegrations per minute (DPM) and wide lines absorbance at 260 nm. Each type of inositol phosphate has two isomers that were tentatively classified as described in the methods. The second isomer of Ins*P*_2_ was eluted at the same position as ATP, and the first isomer of Ins*P*_3_ was eluted at the same position as GTP. In this way, the isomers of radioactive inositol can be easily assigned.

Incubations of SH cells in T‐75 flasks with ^3^H‐myo‐inositol for 24 h were subsequently extracted according to the method described previously and chromatographed at a flow rate of 2.75 mL/ min^−1^. These analyses show, as can be seen in Figure 6 that neuroblastoma cells contain two isomers for each type of inositol phosphate (Ins*P*_1_, Ins*P*_2_, and Ins*P*_3_), the concentrations of Ins*P*_1_ and Ins*P*_2_ being greater than those of InsP3. The isomers were tentatively classified as Ins(1)*P*_1_, Ins(2)*P*_1_, Ins(1,4)*P*_2_, Ins(2,4)*P*_2_, Ins(1,3,4)*P*_3_ and Ins(1,4,5)*P*_3_. Finally, from the radioactivity incorporated into the cells, we were able to calculate the daily phosphorylation rate from ^3^H‐myo‐inositol to Ins*P*1, Ins*P*_2_, and Ins*P*_3_, which is around of 0.05%.

## 4 DISCUSSION

Previous methods used for analysis of inositol phosphates (Azevedo & Saiardi, 2006; Casals et al., 2002; Guse, Greiner, et al., 1993; Letcher et al., 2010; Lorke et al., 2004; Mayr, 1988; Singh & Jiang, 1995) or nucleotides (Chida et al., 2012; Liu et al., 2006; Varik et al., 2017; zur Nedden et al., 2009) do not give overall information of the cellular physiology because they use specific methodologies for different kinds of samples. Despite there being many specific studies on different cell cultures, poor information is available for metabolic and signaling analyses on cell cultures.

The method allows the detection of the main nucleotides in a unique confluent culture of T‐25 flask. Due to the wide range of sensitivity, from 500 picomoles to more than 50 nanomoles, this allows the detection of cell metabolic status (i.e., through the adenylate energy charge or the ATP/ADP ratio) and signaling molecules involved in adrenergic stimulation (cAMP) in a single analytical process. Furthermore, with a prior radioactive incubation with ^3^H‐myo‐inositol at a low level of radioactivity, different isomers of inositol phosphates involved in cell signaling processes can be analyzed in optimal conditions if a T‐75 confluent flask is used. This is useful to be able to evaluate simultaneously signaling pathways and the metabolic condition of the cell culture. At the level of comparative physiology, using a separating solution of 0.4 M HCl, it is easy to evaluate cellular metabolism and the levels of the different isomers of inositol phosphate, molecules of notable evolutionary interest in bird erythrocytes (Villar et al., 2003).

The flow rate proposed of 1 mL min^−1^, better than higher flow rates to obtain a better resolutions and peak shapes, is according to the study of Feng et al. for which the best flow rate for HPLC analysis of nucleotides is 0.8–0.9 mL min^−1^. These authors recommend using a chromatographic column temperature between 30°C and 35°C to obtain better resolution and peak shape (Fenga et al., 2020). Nevertheless, the flow rate has much more effect than temperature. On the other hand, Ledderose et al. recommend maintaining the column compartment at 4°C to prevent nucleotide hydrolysis (Ledderose et al., 2022). We propose a temperature of 16°C–18°C, which is lower than that of the laboratory. At those temperatures, the effect on reducing resolution or increasing nucleotide hydrolysis is very low, and it furthermore helps to solubilize air bubbles that could strongly affect the chromatographic process.

The analyses performed in this study incubating SH‐SY5Y cells with ^3^H‐myo‐inositol show that neuroblastoma cells contain two isomers for each type of inositol phosphate (Ins*P*_1_, Ins*P*_2_, and Ins*P*_3_). In our case, the isomers were tentatively assigned, according to their elution time and by comparison with previously characterized inositols of other neuronal cells (Dean & Moyer, 1987), as Ins(1)*P*_1_, Ins(2)*P*_1_, Ins(1,4)*P*_2_, Ins(2,4)*P*_2_, Ins(1,3,4)*P*_3_ and Ins(1,4,5)*P*_3_. This composition of phosphate inositols opens the possibility that, by cellular activation, one or both isomers of each type of inositol can increase, opening up different possibilities in the cell signaling process.

The acid extraction using 8%–10% TCA resulted very suitably for cell cultures because the amount of TCA needed is low. In previous studies on inositol metabolism in avian erythrocytes (Stephens et al., 1988), TCA extracts are treated with charcoal. Our trials (data not shown) demonstrate that charcoal absorbed nucleotides and then reduced strongly their chromatographic analyses. Therefore, we do not recommend using charcoal if nucleotides are involved in the chromatographic analyses. Then, the samples were neutralized with a low quantity of phosphate buffer. The neutralization with triethanolamine (Lorke et al., 2004) or Tris‐acetate buffer (Chida et al., 2012) is also useful, but in our trials, the phosphate buffer gave us better control in the HPLC analyses proposed. However, as indicated in our results, the amount of phosphate buffer used affects the elution time of the nucleotides monophosphate. This effect could be because free phosphates can form complexes with the nucleotide phosphates in the presence of metal ions (Dudev et al., 2017; Goucher & Taylor, 1964; Mansour et al., 1985) and this negative complex induces a slightly higher interaction between nucleotide and resin. It is important to consider this effect to identify correctly monophosphate nucleotides in cellular analysis.

Chromatographic analyses of nucleotides published previously in different animal tissues showed relative values similar to the data on neuroblastoma cells obtained in the present study. Pani et al. in neuronal tissue obtained ATP/ADP ratios in different mouse encephalic regions from 1,3 in cerebellum or 2,4 in striatum nucleus to 7,1 in cerebral cortex (Pani et al., 2014). Similar ratios were found in whole brain from rat and mouse (1,7 and 2,2 respectively) for Manfredin et al. (Manfredi et al., 2002). In myocardial tissue, Volonté et al. found values of 1,8 in control sample (Volonté et al., 2004).

Recently, Ledderose et al. showed that plasma nucleotide levels are subjected to the extraction procedure that can induce an ATP breakdown, obtaining ATP/ADP values near 1.5 in EDTA‐treated blood samples, but lower than 0.5 when heparin is used (Ledderose et al., 2022). Considering that the ATP/ADP ratio is a good indicator of cellular metabolism, the high ratios found in this study for neuroblastoma cell culture from 4 to 5 using our procedure indicate that the method is very appropriate and useful for a metabolic analysis of the cells.

We have only found a few studies on the specific effect of culture density on cellular metabolism. Bereiter‐Hahn et al. studied the energy metabolism of three related cell types and found that preconfluent densities exhibit relatively low metabolic activity possibly due to the lack of supply with metabolites for catalytic processes (Bereiter‐Hahn et al., 1998). Little changes in pH due to culture density also can affect cell metabolism (Hilal‐Alnaqbi et al., 2013; Michl et al., 2019). This fact is important in studies of cell activation and signaling since different responses would be obtained, depending on the density of the culture. The method has allowed us to characterize the optimal culture conditions for SH‐SY5Y cells. It is also useful for any other type of cultured cells with minor modifications.

Finally, it should be noted that, while not all inositol phosphate isomers are resolved using this method, it is useful for many inositol phosphates of physiological interest present in mammalian cells. Likewise, cGMP elutes in the same position as ADP, but this nucleotide is found at extremely low concentrations and therefore does not influence ADP level.

## 5 CONCLUSION

This method has sufficient sensitivity to simultaneously determine quantitative changes in inositol phosphates and nucleotides in a single confluent T‐75 flask containing only 8 million cells. Moreover, it is possible to analyze the cellular production rate of inositol phosphate in low‐intensity radioactive conditions and assess the accurate levels of cAMP and AMP when a low concentration of B solution is applied.

## AUTHOR CONTRIBUTIONS

**Miquel Riera‐Codina:** conceptualization, writing‐original draft, funding acquisition, writing‐review and editing, formal analysis, project administration, data curation, and resources. **Natalie Chaves Ferreira:** methodology, data curation, formal analysis, investigation, and writing‐review and editing. **Chiara Gatnau‐Civardi:** methodology and investigation.

## FUNDING INFORMATION

This study was supported by funds from the Ministerio de Ciencia, Innovación y Universidades/Agencia Estatal de Investigación (MICIU/AEI)/10.13039/501100011033 (Project: PID2020‐116172RB‐I00).

## CONFLICT OF INTEREST STATEMENT

The authors declare that they have no known competing financial interests or personal relationships that could have appeared to influence the work reported in this paper.

## ETHICS STATEMENT

This study was conducted using cell cultures. There are no ethical certifications.

## IRB STATEMENT

This study did not need an IRB review.

## Data Availability

The data that support the findings of this study are available from the corresponding author, Dr. Miquel Riera Codina, on request.

## References

[phy270419-bib-0001] Azevedo, C. , & Saiardi, A. (2006). Extraction and analysis of soluble inositol polyphosphates from yeast. Nature Protocols, 1, 2416–2422. 17406485 10.1038/nprot.2006.337

[phy270419-bib-0002] Bartlett, G. R. (1980). Phosphate compounds in vertebrate red blood cells. American Zoologist, 20, 103–114.

[phy270419-bib-0003] Bereiter‐Hahn, J. , Münnich, A. , & Woiteneck, P. (1998). Dependence of energy metabolism on the density of cells in culture. Cell Structure and Function, 23, 85–93. 9669036 10.1247/csf.23.85

[phy270419-bib-0004] Berrindge, M. J. , & Irvine, R. F. (1984). Inositol triphosphate, a novel second messenger in cellular signal transduction. Nature, 312, 315–321. 6095092 10.1038/312315a0

[phy270419-bib-0005] Bradbury, D. A. , Simmons, T. D. , Slater, K. J. , & Crouch, S. P. M. (2000). Measurement of ADP: ATP ratio in human leukaemic cell lines can be used as an indicator of cell viability, necrosis and apoptosis. Journal of Immunological Methods, 240, 79–92. 10854603 10.1016/s0022-1759(00)00178-2

[phy270419-bib-0006] Burton, A. , Hu, X. , & Saiardi, A. (2009). Are inositol pyrophosphates signalling molecules? Journal of Cellular Physiology, 220, 8–15. 19326391 10.1002/jcp.21763

[phy270419-bib-0007] Casals, I. , Villar, J. L. , & Riera‐Codina, M. (2002). A straightforward method for analysis of highly phosphorylated inositols in blood cells by high‐performance liquid chromatography. Analytical Biochemistry, 300, 69–76. 11743693 10.1006/abio.2001.5449

[phy270419-bib-0008] Chatree, S. , Thongmaen, N. , Tantivejkul, K. , Sitticharoon, C. , & Vucenik, I. (2020). Role of inositol and inositol phosphate in energy metabolism. Molecules, 25, 5079. 33139672 10.3390/molecules25215079 PMC7663797

[phy270419-bib-0009] Chida, J. , Yamane, K. , Takei, T. , & Kido, H. (2012). AN efficient extraction method for quantification of adenosine triphosphate in mammalian tissue and cells. Analytica Chimica Acta, 727, 8–12. 22541816 10.1016/j.aca.2012.03.022

[phy270419-bib-0010] Cohn, M. , & Hughes, T. R., Jr. (1960). Phosphorus magnetic resonance spectra of adenosine di‐ and triphosphate. I. Effect of pH. Journal of Biological Chemistry, 235, 3250–3253. 13694477

[phy270419-bib-0011] Dean, M. N. , & Moyer, J. D. (1987). Separation of multiple isomers of inositol phosphate formed in GH3 cells. Biochemical Journal, 242, 361–366. 3109388 10.1042/bj2420361 PMC1147713

[phy270419-bib-0012] Dudev, T. , Grauffel, C. , & Lim, C. (2017). How native and alien metal cations bind ATP:M implications for lithium as a therapeutics agent. Scientific Reports, 7, 42377. 28195155 10.1038/srep42377 PMC5307966

[phy270419-bib-0013] Eckmann, L. , Rudolf, M. T. , Ptasznik, A. , Schultz, C. , Jiang, T. , Wolfson, N. , Tsien, R. , Fierer, J. , Shears, S. B. , Kagnoff, M. F. , & Traynor‐Kaplan, A. E. (1997). D‐myo‐inositol 1,4,5,6‐tetrakisphosphate produced in human intestinal epithelial cells in response to salmonella invasion inhibits phosphoinositide 3‐kinase signaling pathways. Proceedings of the National Academy of Sciences of the United States of America, 94, 14456–14460. 9405634 10.1073/pnas.94.26.14456 PMC25019

[phy270419-bib-0014] Fenga, J.‐H. , Weia, K.‐Z. , Gaoa, J.‐P. , & Xub, X. (2020). Determination of adenosine phosphates in mouse myocardium tissue by HPLC with UV detection and using porous graphite carbon column. Journal of Chromatography. B, Biomedical Sciences and Applications, 1145, 122110. 10.1016/j.jchromb.2020.122110 32315974

[phy270419-bib-0015] Goucher, C. R. , & Taylor, J. F. (1964). Compounds of ferric iron with adenosine triphosphate and other nucleoside phosphates. The Journal of Biological Chemistry, 239, 2251–2255. 14209955

[phy270419-bib-0016] Guse, A. H. , Greiner, E. , Emmrich, F. , & Brand, K. (1993). Mass change of inositol 1,3,4,5,6‐pentakisphosphate and inositol hexakisphosphate during cell cycle progression in rat thymocytes. Journal of Biological Chemistry, 268, 7129–7133. 8463248

[phy270419-bib-0017] Guse, A. H. , Roth, E. , & Emmrich, F. (1993). Intracellular Ca^2+^ pools in Jurkat T‐lymphocytes. The Biochemical Journal, 291, 447–451. 8484725 10.1042/bj2910447 PMC1132546

[phy270419-bib-0018] Hilal‐Alnaqbi, A. , Hu, A. Y. C. , Zhang, Z. , & Al‐Rubeai, M. (2013). Growth, metabolic activity, and productivity of immobilized and freely suspended CHO cells in perfusion culture. Biotechnology and Applied Biochemistry, 60, 436–445. 23701045 10.1002/bab.1103

[phy270419-bib-0019] Ho, M. W. Y. , Yang, X. , Carew, M. A. , Zhang, T. , Hua, L. , Kwon, Y.‐U. , Chung, S.‐K. , Adelt, S. , Vogel, G. , Riley, A. M. , Potter, B. V. L. , & Shears, S. B. (2002). Regulation of ins(3,4,5,6,)P4 signaling by reversible kinase/phosphatase. Current Biology, 12, 477–482. 11909533 10.1016/s0960-9822(02)00713-3

[phy270419-bib-0020] Horstman, D. A. , Takemura, H. , & Putney, J. W., Jr. (1988). Formation and metabolism of [^3^H]inositol phosphates in AR42J pancreatoma cells. Journal of Biological Chemistry, 263, 15297–15303. 2459122

[phy270419-bib-0021] Irvine, R. F. (2005). Inositide evolution ‐ towards turtle domination? The Journal of Physiology, 566, 295–300. 15860522 10.1113/jphysiol.2005.087387 PMC1397712

[phy270419-bib-0022] Irvine, R. F. , & Schell, M. J. (2001). Back in the water: The return of the inositol phosphate. Nature Reviews Molecular Cell Biology, 2, 327–338. 11331907 10.1038/35073015

[phy270419-bib-0023] Isaacks, R. E. , & Harkness, D. R. (1980). Erythrocyte organic phosphates and hemoglobin function in birds, reptiles and fishes. American Zoologist, 20, 115–129.

[phy270419-bib-0024] Jia, Y. , Subramanian, K. K. , Erneux, C. , Pouillon, V. , Hattori, H. , Jo, H. , You, J. , Zhu, D. , Schurmans, S. , & Luo, H. R. (2007). Inositol 1,3,4,5‐tetrakisphosphate negatively regulates chemoattractant‐elicited PtdIns(3,4,5)P3 signaling in neutrophils. Immunity, 27, 453–467. 17825589 10.1016/j.immuni.2007.07.016 PMC2084373

[phy270419-bib-0025] King, J. , Keim, M. , Teo, R. , Weening, K. E. , Kapur, M. , MacQuillan, K. , Ryves, J. , Rogers, B. , Dalton, E. , Williams, R. S. B. , & Harwood, A. J. (2010). Genetic control of lithium sensitivity and regulation of inositol biosynthetic genes. PLoS One, 5, e11151. 20567601 10.1371/journal.pone.0011151 PMC2887444

[phy270419-bib-0026] Kovalevich, J. , Santerre, M. , & Langford, D. (2021). Chapter 2: Considerations for the use of SH‐SY5Y neuroblastoma cells in neurobiology. In Neuronal cell culture—Methods and protocols (Vol. 2311, 2nd ed., pp. 9–23). Human Press. 10.1007/978-1-0716-1437-2_2 34033074

[phy270419-bib-0027] Ledderose, C. , Valsami, E.‐A. , & Junger, W. G. (2022). Optimized HPLC method to elucidate the complex purinergic signaling dynamics that regulate ATP, ADP, AMP, and adenosine levels in human blood. Purinergic Signalling, 18, 223–239. 35132577 10.1007/s11302-022-09842-w PMC9123122

[phy270419-bib-0028] Letcher, A. J. , Schell, M. J. , & Irvine, R. F. (2008). Do mammals make all their own inositol hexakisphosphate? The Biochemical Journal, 416, 263–270. 18684107 10.1042/BJ20081417 PMC2605958

[phy270419-bib-0029] Letcher, A. J. , Schell, M. J. , & Irvine, R. F. (2010). A femtomole‐sensitivity mass assay for inositol hexakisphosphate. Methods in Molecular Biology, 645, 61–71. 20645181 10.1007/978-1-60327-175-2_4

[phy270419-bib-0030] Liu, H. , Jiang, Y. , Luo, Y. , & Jiang, W. (2006). A simple and rapid determination of ATP, ADP and AMP concentration in pericarp tissue of litchi fruit by high performance liquid chromatography. Food Technology and Biotechnology, 44, 531–534.

[phy270419-bib-0031] Lorke, D. E. , Gustke, H. , & Mayr, G. W. (2004). An optimized fixation and extraction technique for high resolution of inositol phosphate signals in rodent brain. Neurochemical Research, 29, 1887–1896. 15532545 10.1023/b:nere.0000042216.86633.71

[phy270419-bib-0032] Loss, O. , Wu, C. T. , Riccio, A. , & Saiardi, A. (2013). Modulation of inositol polyphosphate levels regulates neuronal differentiation. Molecular Biology of the Cell, 24, 2981–2989. 23864704 10.1091/mbc.E13-04-0198 PMC3771958

[phy270419-bib-0033] Maffucci, T. , & Falasca, M. (2020). Signalling properties of inositol polyphosphates. Molecules, 25, 5281. 33198256 10.3390/molecules25225281 PMC7696153

[phy270419-bib-0034] Manfredi, G. , Yang, L. , Gajewski, C. D. , & Mattiazzi, M. (2002). Measurements of ATP in mammalian cells. Methods, 26, 317–326. 12054922 10.1016/S1046-2023(02)00037-3

[phy270419-bib-0035] Mansour, A. N. , Thompsonq, C. , Theil, E. C. , Chasteen, N. D. , & Sayers, D. E. (1985). Fe(II1) ATP complexes. Models for ferritin and other polynuclear iron complexes with phosphate. Journal of Biological Chemistry, 260, 7975–7979. 2989269

[phy270419-bib-0036] Matejuk, A. , & Shamsuddin, A. (2010). IP6 in cancer therapy: Past, present and future. Current Cancer Therapy Reviews, 6, 1–12.

[phy270419-bib-0037] Mayr, G. W. (1988). A novel metal‐dye detection system permits picomolar‐range h.p.l.c. analysis of inositol polyphosphate from non‐radioactively labelled cell or tissue specimens. Biochemical Journal, 254, 585–591. 3178774 10.1042/bj2540585 PMC1135118

[phy270419-bib-0038] Michell, R. H. (2008). Inositol derivatives: Evolution and function. Nature Reviews Molecular Cell Biology, 9, 151–161. 18216771 10.1038/nrm2334

[phy270419-bib-0039] Michl, J. , Park, K. C. , & Swietach, P. (2019). Evidence‐based guidelines for controlling pH in mammalian live‐cells culture systems. Communications Biology, 2, 144. 31044169 10.1038/s42003-019-0393-7 PMC6486606

[phy270419-bib-0040] Mountford, J. C. , Bunce, C. M. , French, P. R. , Michell, R. H. , & Brown, G. (1994). Intracellular concentration of inositol, glycerophosphoinositol and inositol pentakisphosphate increase during haemopoietic cell differentiation. Biochimica et Biophysica Acta, 1222, 101–108. 7514443 10.1016/0167-4889(94)90030-2

[phy270419-bib-0041] Pani, A. K. , Jiao, Y. , Sample, K. J. , & Smeyne, R. J. (2014). Neurochemical measurement of adenosine in discrete brain regions of five strains of inbred mice. PlosOne, 9, e92422. 10.1371/journal.pone.0092422 PMC3958516 24642754

[phy270419-bib-0042] Piccolo, E. , Vignati, S. , Maffucci, T. , Innominato, P. F. , Riley, A. M. , Potter, B. V. L. , Pandolfi, P. P. , Broggini, M. , Iacobelli, S. , Innocent, P. , & Falasca, M. (2004). Inositol pentakisphosphate promote apoptosis through the PI 3‐K/Akt pathway. Oncogene, 23, 1754–1765. 14755253 10.1038/sj.onc.1207296

[phy270419-bib-0043] Poulsen, J. C. , Caspersen, C. , Mathiasen, D. , East, J. M. , Tunwell, R. E. , Lai, F. A. , Maeda, N. , Mikoshiba, K. , & Treiman, M. (1995). Thapsigargin‐sensitive Ca(^2+^)‐ATPases account for Ca^2+^ uptake to inositol‐1,4,5‐triphosphate‐sensitive and caffeine‐sensitive Ca^2+^ stores in adrenal chromaffin cells. Biochemical Journal, 307, 749–758. 7741706 10.1042/bj3070749 PMC1136714

[phy270419-bib-0044] Prete, A. D. , Zaccagnino, P. , di Paola, M. , Saltarella, M. , Celis, C. O. , Nico, B. , Santoro, G. , & Lorusso, M. (2008). Role of mitochondria and reactive oxygen species in dendritic cell differentiation and function. Free Radical Biology and Medicine, 44, 1443–1451. 18242195 10.1016/j.freeradbiomed.2007.12.037

[phy270419-bib-0045] Renström, E. , Ivarsson, R. , & Shears, S. B. (2002). Ins(3,4,5,6)P4 inhibits insulin granulate acidification and fusogenic potential. Journal of Biological Chemistry, 277, 26717–26720. 12055181 10.1074/jbc.C200314200

[phy270419-bib-0046] Riera, M. , Fuster, J. F. , & Palacios, L. (1991). Role of erythrocyte organic phosphates in blood oxygen transport in anemic quail. The American Journal of Physiology, 260, R798–R803. 2012250 10.1152/ajpregu.1991.260.4.R798

[phy270419-bib-0047] Rossier, M. F. , Bird, G. S. , & Putney, J. W., Jr. (1991). Subcellular distribution of the calcium storing inositol 1,4,5‐triphosphate‐sensitive organelle in rat liver. Possible linked to the plasma membrane through the actin microfilaments. Biochemical Journal, 274, 643–650. 1849402 10.1042/bj2740643 PMC1149960

[phy270419-bib-0048] Sharp, A. H. , Nucifora, F. C., Jr. , Blondel, O. , Sheppard, C. A. , Zhang, C. , Snyder, S. H. , Russell, T. J. , Ryugo, D. K. , & Ross, C. A. (1999). Differential cellular expression of isoforms of inositol 1,4,5‐triphosphate receptors in neurons and glia in brain. Journal of Comparative Neurology, 406, 207–220. 10096607

[phy270419-bib-0049] Singh, A. K. , & Jiang, Y. (1995). Quantitative chromatographic analysis of inositols phospholipids and related compounds. Journal of Chromatography. B, Analytical Technologies in the Biomedical and Life Sciences, 671, 255–280. 10.1016/0378-4347(94)00558-m 8520695

[phy270419-bib-0050] Stephens, L. , Hawkins, P. T. , Carter, N. , Chahwala, S. B. , Morris, A. J. , Whetton, A. D. , & Downes, P. C. (1988). L‐myo‐inositol 1,4,5,6‐tetrakisphosphate is present in both mammalian and avian cells. Journal of Biochemistry, 249, 271–282. 10.1042/bj2490271 PMC1148694 3342011

[phy270419-bib-0051] van Acker, K. , Bautmans, B. , Bultynck, G. , Maes, K. , Weidema, A. F. , de Smet, P. , Parys, J. B. , de Smedt, H. , Missiaen, L. , & Callewaert, G. (2000). Mapping of IP3‐mediated Ca^2+^ signals in single human neuroblastoma SH‐SY5Y cells: Cell volume shaping the Ca^2+^ signal. Journal of Neurophysiology, 83, 1052–1057. 10669516 10.1152/jn.2000.83.2.1052

[phy270419-bib-0052] van Delden, C. , Foti, M. , Lew, D. P. , & Krause, K. H. (1993). Ca^2+^ and Mg^2+^ regulation of inositol 1,4,5‐triphosphate biding in myeloid cells. Journal of Biological Chemistry, 268, 12443–12448. 8389758

[phy270419-bib-0053] Varik, V. , Oliveira, S. R. A. , Hauryliuk, V. , & Tenson, T. (2017). HPLC‐based quantification of bacterial housekeeping nucleotides and alarmone messengers ppGpp and pppGpp. Scientific Reports, 7, 11022. 28887466 10.1038/s41598-017-10988-6 PMC5591245

[phy270419-bib-0054] Villar, J. L. , Puigbò, P. , & Riera‐Codina, M. (2003). Analysis of highly phosphorylate inositol in avian and crocodilian erythrocytes. Comparative Biochemistry and Physiology. Part B, Biochemistry & Molecular Biology, 135, 169–175. 10.1016/s1096-4959(03)00077-0 12781983

[phy270419-bib-0055] Volonté, M. G. , Yulna, G. , Quiroga, P. , & Consolini, A. E. (2004). Development of an HPLC method for determination of metabolic compounds in myocardial tissue. Journal of Pharmaceutical and Biomedical Analysis, 35, 647–653. 15137992 10.1016/j.jpba.2004.02.002

[phy270419-bib-0056] Wilcox, R. A. , Strupish, J. , & Nahorski, S. R. (1996). Quantal calcium release in electropermeabilized SH‐SY5Y neuroblastoma cells perfused with myo‐inositol 1,4,5‐triphosphate. Cell Calcium, 20, 243–255. 8894271 10.1016/s0143-4160(96)90030-5

[phy270419-bib-0057] Xie, W. , Kaetzel, M. A. , Bruzik, K. S. , Dedman, J. R. , Shears, S. B. , & Nelson, D. J. (1996). Inositol 3,4,5,6‐tretakisphosphate inhibits the calmodulin‐dependent protein kinase II‐activated chloride conductance in T84 colonic epithelial cells. Journal of Biological Chemistry, 271, 14092–14097. 8662902 10.1074/jbc.271.24.14092

[phy270419-bib-0058] Yang, X. , Rudolf, M. , Carew, M. A. , Yoshida, M. , Nerreter, V. , Riley, A. M. , Chung, S.‐K. , Bruzik, K. S. , Potter, B. V. L. , Schultz, C. , & Shears, S. B. (1999). Inositol‐1,3,4 triphosphate acts in vivo as a specific regulator of cellular signaling by inositol‐3,4,5,6 tetrakisphosphate. Journal of Biological Chemistry, 274, 18973–18980. 10383396 10.1074/jbc.274.27.18973

[phy270419-bib-0059] zur Nedden, S. , Eason, R. , Doney, A. S. , & Frenguelli, B. G. (2009). An ion‐pair reversed‐phase HPLC method for determination of fresh tissue adenine nucleotides avoiding freeze‐thaw degradation of ATP. Analytical Biochemistry, 388, 108–114. 19233119 10.1016/j.ab.2009.02.017

